# Renal carcinoma/kidney progenitor cell chimera organoid as a novel tumorigenesis gene discovery model

**DOI:** 10.1242/dmm.028332

**Published:** 2017-12-01

**Authors:** Qi Xu, Sanna Junttila, Andreas Scherer, Khem Raj Giri, Oona Kivelä, Ilya Skovorodkin, Juha Röning, Susan E. Quaggin, Hans-Peter Marti, Jingdong Shan, Anatoly Samoylenko, Seppo J. Vainio

**Affiliations:** 1 Biocenter Oulu, Laboratory of Developmental Biology, InfoTech Oulu, Center for Cell Matrix Research, Faculty of Biochemistry and Molecular Medicine, Oulu University, FI-90014 Oulu, Finland; 2 Spheromics, FI-81100 Kontiolahti, Finland; 3 ValiFinn, FI-90220 Oulu, Finland; 4 Department of Computer Science and Engineering, University of Oulu, FI-90014 Oulu, Finland; 5 Feinberg Cardiovascular Research Institute, Division of Medicine-Nephrology, Northwestern University, Feinberg School of Medicine, Chicago, IL 60611, USA; 6 Department of Clinical Medicine, University of Bergen, N-5020 Bergen, Norway

**Keywords:** Renal cell carcinoma, RCC, Nephrogenesis, Gene expression, siRNA

## Abstract

Three-dimensional (3D) organoids provide a new way to model various diseases, including cancer. We made use of recently developed kidney-organ-primordia tissue-engineering technologies to create novel renal organoids for cancer gene discovery. We then tested whether our novel assays can be used to examine kidney cancer development. First, we identified the transcriptomic profiles of quiescent embryonic mouse metanephric mesenchyme (MM) and of MM in which the nephrogenesis program had been induced *ex vivo*. The transcriptome profiles were then compared to the profiles of tumor biopsies from renal cell carcinoma (RCC) patients, and control samples from the same kidneys. Certain signature genes were identified that correlated in the developmentally induced MM and RCC, including components of the caveolar-mediated endocytosis signaling pathway. An efficient siRNA-mediated knockdown (KD) of *Bnip3*, *Gsn*, *Lgals3*, *Pax8*, *Cav1*, *Egfr* or *Itgb2* gene expression was achieved in mouse RCC (Renca) cells. The live-cell imaging analysis revealed inhibition of cell migration and cell viability in the gene-KD Renca cells in comparison to Renca controls. Upon siRNA treatment, the transwell invasion capacity of Renca cells was also inhibited. Finally, we mixed E11.5 MM with yellow fluorescent protein (YFP)-expressing Renca cells to establish chimera organoids. Strikingly, we found that the *Bnip3-*, *Cav1-* and *Gsn*-KD Renca-YFP+ cells as a chimera with the MM in 3D organoid rescued, in part, the RCC-mediated inhibition of the nephrogenesis program during epithelial tubules formation. Altogether, our research indicates that comparing renal ontogenesis control genes to the genes involved in kidney cancer may provide new growth-associated gene screens and that 3D RCC-MM chimera organoids can serve as a novel model with which to investigate the behavioral roles of cancer cells within the context of emergent complex tissue structures.

## Abstract (precis)

**Editor’s Choice:** Chimeras between embryonic kidney cells and renal carcinoma cells serve as a novel model to assay the roles of co-regulated genes in kidney development and renal carcinogenesis.

## INTRODUCTION

The ability to model diseases such as cancer by targeting candidate genes in embryonic stem cells and by making *in vivo* mouse models from multipotent cells revolutionized pathogenesis studies (Lim et al., 2016). Recently, it has also become possible to reprogram normal and dysfunctional adult cells into stem cells and to develop organoids that form specific cell lineages. These complex organ-like cell aggregates provide a way to model tumorigenesis *ex vivo* (Lovitt et al., 2016).

Cancer organoid models should offer the possibility to identify the initial steps of tumorigenesis. We propose that the genes responsible for this process can be found among normal developmental regulators. Indeed, processes such as cell proliferation, cell differentiation, cell migration and apoptosis are all involved during normal organogenesis but are associated with malignancy as well. An accumulation of mutational load in the normal developmental signaling pathways may eventually dysregulate and/or reactivate the pathways in adults (Ma et al., 2010; Aiello and Stanger, 2016). Such changes are considered to occur in the kidney (Potter et al., 2010; Sültmann et al., 2005; Yang et al., 2014), where the Wnt, Notch and Sonic hedgehog (SHH) growth factor (GF) pathways (Katoh, 2007; Polakis, 2000; Sjölund et al., 2011; Sun et al., 2009) regulate cell division and cell differentiation in a controlled manner but, when ectopically activated in the adult, they promote malignant growth (Dormoy et al., 2012; Ohnishi et al., 2014).

The fact that ontogenesis and oncogenesis involve related genetic programs is also reflected at the cellular level in processes such as epithelial-mesenchymal transition (EMT) and mesenchymal-epithelial transition (MET) (Thiery et al., 2009). Both are necessary for normal renal development. In the context of malignancy, EMT activation converts benign cells into more invasive ones (Kalluri and Weinberg, 2009; Pang et al., 2011; Rhim et al., 2012), whereas MET is linked to the acquired capacity of the cells to colonize ectopic lesions in metastasis (Yao et al., 2011). These multistep processes represent yet another similarity between developmental control and tumorigenesis. In both cases, GF-promoted angiogenesis is essential to ensure blood supply.

Renal cell carcinoma (RCC) accounts for around 90% of all kidney cancers (Ljungberg et al., 2011). Smoking, obesity, certain chemicals and genetic factors are implicated in RCC promotion (Chow et al., 2010). Chemotherapy for RCC is still very limited. Angiogenesis inhibitors are initially effective, but lose their efficacy because resistance develops (van der Mijn et al., 2014). The small-interfering RNAs (siRNAs) are considered promising anti-cancer compounds (Burnett and Rossi, 2012; Castanotto and Rossi, 2009; Sakurai et al., 2013). They are also useful tools to screen candidate oncogenes and their targets in cell transformation.

In light of the similarities between kidney development and carcinogenesis, we assayed whether some developmental genes may be relevant in kidney malignancy. We began by comparing gene expression between human RCC and experimentally induced mouse nephrogenesis, and identified the genes whose expression was changed in both models. To narrow down our research, we identified the pathways of the genes that showed a markedly changed expression both during kidney development and carcinogenesis. Based on our pathway analysis and published research data (Sohn et al., 2016), we selected the caveolin-related genes for further investigation. We found that siRNA-mediated silencing of BCL2/adenovirus E1B 19 kDa protein-interacting protein 3 (*Bnip3*), gelsolin (*Gsn*) and caveolin-1 (*Cav1*) diminished the growth and motility of mouse RCC (Renca) cells. Next, we investigated whether these cell activities could be modeled in the *ex vivo* chimeras between Renca cells and the kidney progenitor organoids as well. We developed a three-dimensional (3D) co-culture method that makes it possible to study the cross-interactions between embryonic and transformed cells under conditions in which expression of certain genes is inhibited by siRNA treatment. In this model, knockdown of *Bnip3*, *Cav1* or *Gsn* in yellow fluorescent protein (YFP)-expressing Renca cells partially rescued the RCC-mediated inhibition of the nephrogenesis program. Together, the comparative analysis of the ontogenesis and oncogenesis control genes and their functional analysis in a novel chimera organoid between kidney RCC tumor cells and kidney progenitors illustrate the power of the 3D setups for functional discovery of tumorigenic genes.

## RESULTS

### Identification of putative growth and differentiation control genes by comparing the transcriptomes between human ccRCC patients and primary mouse nephron progenitors

Given the similarities between cancer and developmental processes, comparative gene expression profiling may serve to identify relevant candidate factors behind dysregulated cell division and cell differentiation in cancer. To test these ideas, we took advantage of the classic metanephric mesenchyme (MM) kidney-tubule induction model (Junttila et al., 2015) and identified the transcriptome of the non-induced and induced MM (E11.5; 0 h and 96 h, respectively). The gene expression profiles were then compared with the human clear-cell RCC (ccRCC)-generated transcriptome data.

The profiling screen identified 1616 differentially expressed genes in the induced MMs when compared to the non-induced ones (Table S1). When these expression profiles were further compared to those of the human ccRCC cohort (Eikrem et al., 2016), 930 genes were found whose expression was changed simultaneously in both data sets (Table S2). These 930 genes were classified into four groups (Table S2). The majority of the genes showed a similar pattern of expression changes in carcinogenesis and normal development: 534 were upregulated upon MM tubule induction and were abundant also in the ccRCC cohort, whereas only 69 genes were downregulated in both the ccRCC and the MM samples. The rest of the genes demonstrated the opposite behavior: 273 genes were upregulated in the induced MMs but downregulated in the ccRCC samples; in the reverse case, there were 56 genes whose expression was downregulated in the MMs but upregulated in the ccRCC cohort samples (Table S2). Based on these findings, we conclude that the induction of MM and the formation of ccRCC possibly share gene regulation and biological signaling.

**Fig. 1.**
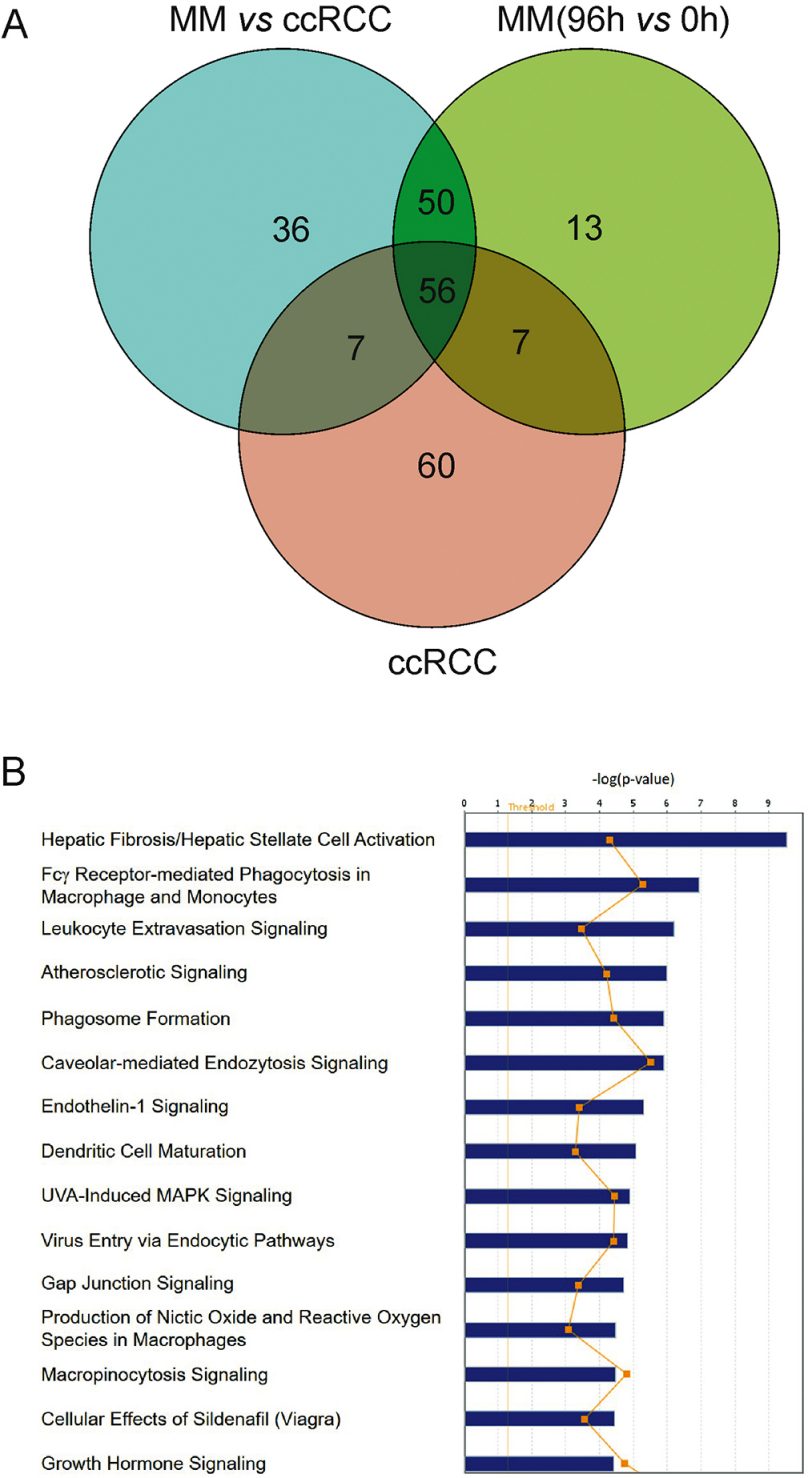
**Ingenuity pathway analysis of the genes enriched in both 96-h-induced MMs and human ccRCC.** (A) Venn diagram shows the number of pathways identified by Ingenuity pathway analysis for three groups of genes: regulated in the kidney development model [MM (96 versus 0 h)], regulated in renal carcinoma patients (ccRCC), and genes simultaneously regulated in both groups. The full pathway list is shown in Table S3. (B) Ingenuity pathway analysis of the genes with differential expression in both the ccRCC and the 96-h-induced MMs showing significantly modulated pathways (*P*<0.05). The 15 top pathways with significant changes are shown. The *P*-value for each pathway is indicated by the bar and is expressed as −1 times the log of the *P*-value. The line connecting the orange squares represents the ratio of the number of genes in a given pathway that meet the cutoff criteria to the total number of genes which belong to the pathway.

The identified non-redundant genes with expression changes of twofold or higher and a *P*-value of <0.05 were subjected to Ingenuity pathway analysis. The Venn diagram (Fig. 1A) shows the number of identified pathways in each of the following groups: pathways significantly involved in the process of ccRCC formation (marked ccRCC), pathways induced in kidney development (marked MM; 96 h versus 0 h), and pathways of genes simultaneously changed in kidney development and in renal carcinogenesis (marked MM versus ccRCC) (the complete lists of pathways are shown in Table S3).

As can be seen from the diagram (Fig. 1A), 56 pathways were shared between all analyzed groups. In order to find a way to prioritize pathways for further analysis, we checked each group using a much stricter significance threshold [−log(*P*-value)>4]. Using this threshold, we found only six pathways that were simultaneously regulated in all three analyzed groups: atherosclerosis signaling, caveolar-mediated endocytosis signaling, dendritic cell maturation, Fcγ-receptor-mediated phagocytosis in macrophages and monocytes, hepatic fibrosis/hepatic stellate cell activation, and leukocyte extravasation signaling. Several of these pathways (such as hepatic fibrosis) require regulation of multiple genes that have very broad functions and are involved in multiple different processes, whereas others were more specifically related to tumor development.

For further analysis, we preferred to concentrate on a pathway already known to be involved in carcinogenesis. In particular, caveolar-mediated endocytosis signaling, which is one of the top signaling pathways found in ccRCC and induced MM, is known to be responsible for cancer heterogeneity when deregulated (Martinez-Outschoorn et al., 2015). This pathway also showed the highest ratio of the number of genes in a given pathway that meet the cutoff criteria to the total number of genes that belong to the pathway in our study (Fig. 1B).

**Fig. 2.**
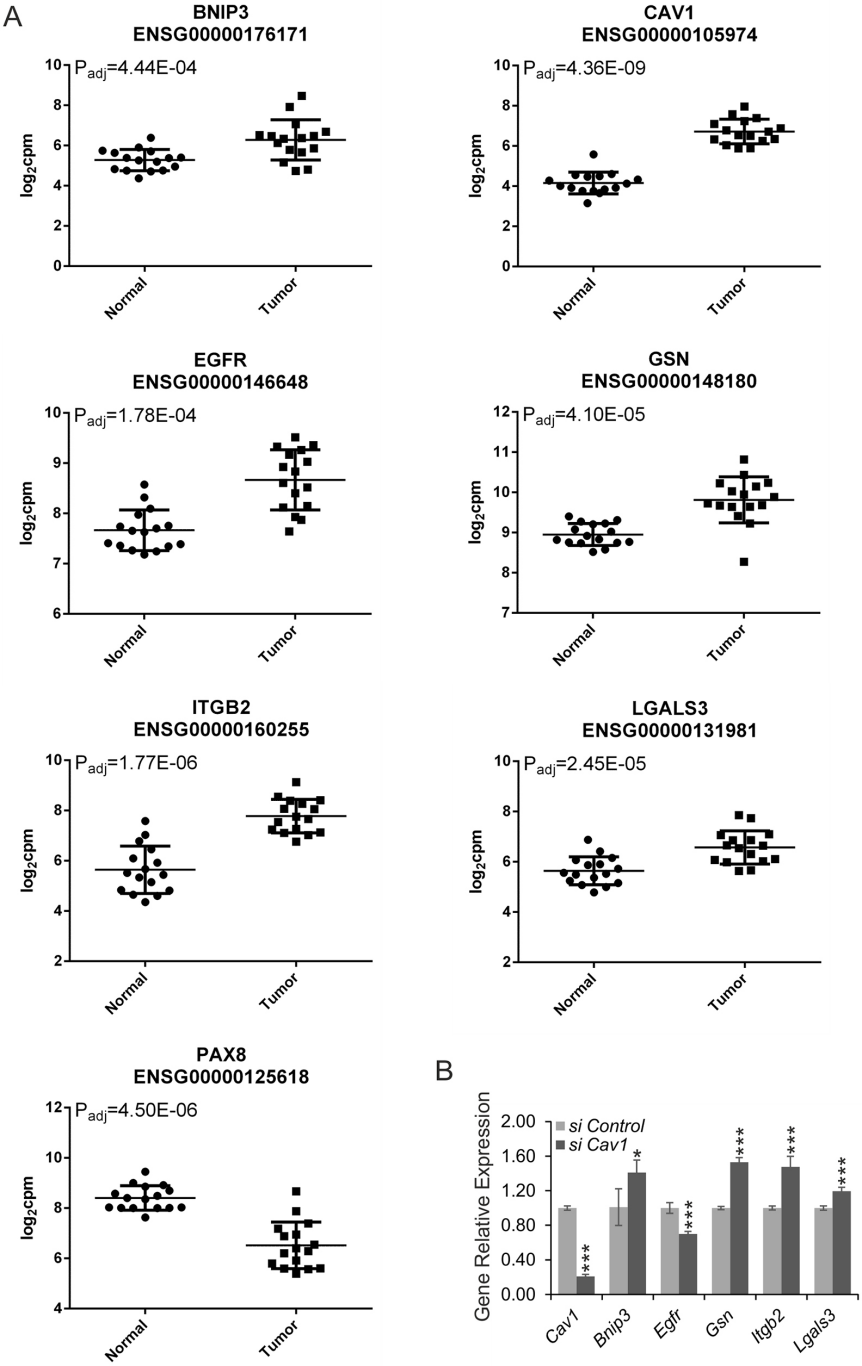
**Gene expression profile in kidney tumors.** (A) The genes that are differentially expressed in both 96-h-induced MMs and human ccRCC were selected after pathway analysis. The gene expression microarray values for ccRCC samples and normal tissue from the same patient (*n*=16) are shown. Ensembl gene numbers (beginning ENSG) are also given. (B) Gene expression profiles after 48 h *Cav1*-siRNA treatment (*si Cav1*), relative to control-siRNA treatment (*si Control*), in Renca cells. The results are presented as means±s.d., and data from three independent experiments are shown. *P<0.05 and ***P<0.001 compared by *t*-test with control siRNA-transfected Renca cells.

Among the caveolar-mediated endocytosis signaling genes induced in the MM that were also abundant in the ccRCC samples were galectin-3 (*Lgals3*), *Gsn*, *Cav1*, epidermal growth factor receptor (*Egfr*) and integrin beta2 (*Itgb2*) (Fig. 2A). For the functional studies, we selected the signaling genes mentioned above and *Bnip3*, which is upregulated in the ccRCC cohort but downregulated in induced MM. It has previously been shown that *Bnip3* expression increases upon *Cav1* downregulation in stromal fibroblasts (Martinez-Outschoorn et al., 2010). Paired box 8 (*Pax8*), a gene encoding a carcinogenesis-related transcription factor necessary for kidney development (Sharma et al., 2015), behaves in the opposite manner to *Bnip3* in the comparative gene array, being downregulated in the ccRCC cohort but upregulated in the MM undergoing differentiation. We also analyzed expression of *Lgals3*, *Gsn*, *Cav1*, *Egfr*, *Itgb2*, *Bnip3* and *Pax8* using publically available data from 144 biopsies (77 ccRCC, 77 normal) in Gene Expression Omnibus (GEO) database GSE53757. The directions of the expression changes for these genes in a bigger GEO data set were mostly the same as in our smaller collection of human ccRCC samples (Table S4).

### siRNAs as putative ‘drugs’ to compromise RCC behavior in model cells *in vitro*

After selecting candidate genes for closer study, we chose siRNAs as the tools to screen for functional knockdown of these genes. The efficiency of siRNA-mediated downregulation of gene expression was studied in mouse RCC (Renca) cells after transfection with 50 nM siRNA in 96-well plates by reverse transcription–quantitative real-time PCR (RT-qPCR) (48, 72 and 96 h) and was found to be in the range of 83-97% inhibition (Fig. S1A). We also analyzed protein levels for two genes (*Cav1* and *Gsn*) at the same time points by western blot (Fig. S1B), and the protein data was similar to RNA data. In addition, we showed that lowering expression of *Cav1* in Renca cells slightly but significantly increased the mRNA levels of *Bnip3*, *Gsn*, *Itgb2* and *Lgals3*, whereas *Egfr* expression was decreased (Fig. 2B).

**Fig. 3.**
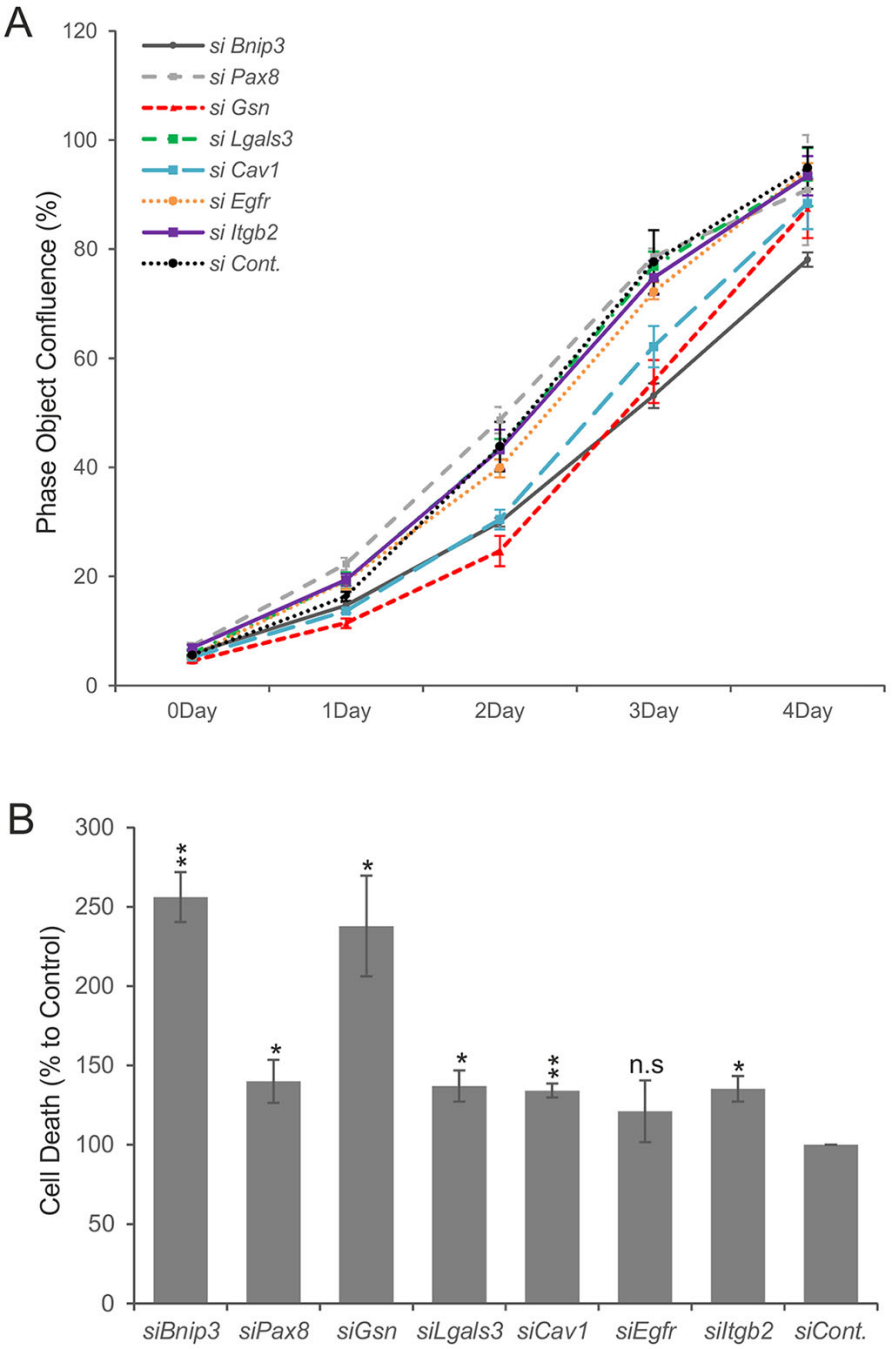
**Effect of siRNA transfection on the viability of Renca cells.** (A) Proliferation assay; (B) cell death. Renca cells were pre-transfected with various siRNA oligonucleotides (*si*; 50 nM each) for 2 days, seeded on 96-well plates at a density of 3000 cells/well and imaged by IncuCyte. Cell proliferation was presented as a percentage of the confluent area. To study cell death, SYTOX Green was added, both phase contrast and the green channel were measured, and the percentage of the green area was calculated. The results are presented as means±s.d. Data from three independent experiments are shown. *P<0.05 and **P<0.01 compared by *t*-test with control siRNA-transfected Renca cells. n.s., not significant.

In order to depict possible changes in the Renca cells after siRNA transfections, we first analyzed Renca cell proliferation by live imaging using IncuCyte during a 4-day culture time. The results showed that the siRNA-mediated silencing of the *Bnip3*, *Gsn* and *Cav1* genes notably reduced Renca cell proliferation in comparison to the controls, whereas the other tested siRNAs, namely for *Lgals3*, *Pax8*, *Egfr* and *Itgb2*, did not influence cell growth (Fig. 3A).

To assay whether siRNA-mediated gene silencing would have an impact on cell viability, the cells were stained with SYTOX Green nuclear acid dye, and the percentage of dead cells at 24 h after cell plating was illustrated. Silencing of the expression of all assayed genes, except for *Egfr*, promoted cell death as compared to the scrambled-siRNA-transfected controls, although to different extents.

The siRNA targeting of the *Bnip3* and the *Gsn* mRNA species turned out to be the most effective with respect to cytotoxicity. In these cases, cell death was induced by around 2.5-fold and 2.4-fold, respectively (Fig. 3B). Thus, siRNA-mediated knockdown of a panel of the genes regulated upon nephrogenesis activation in the MM and that are highly expressed also in RCC cells suggested that the strategy may be useful to identify novel growth-control-associated genes in RCC.

### siRNA-mediated silencing of certain developmental and RCC-associated genes inhibits cell migration, invasion and colony-forming ability *in vitro*

**Fig. 4.**
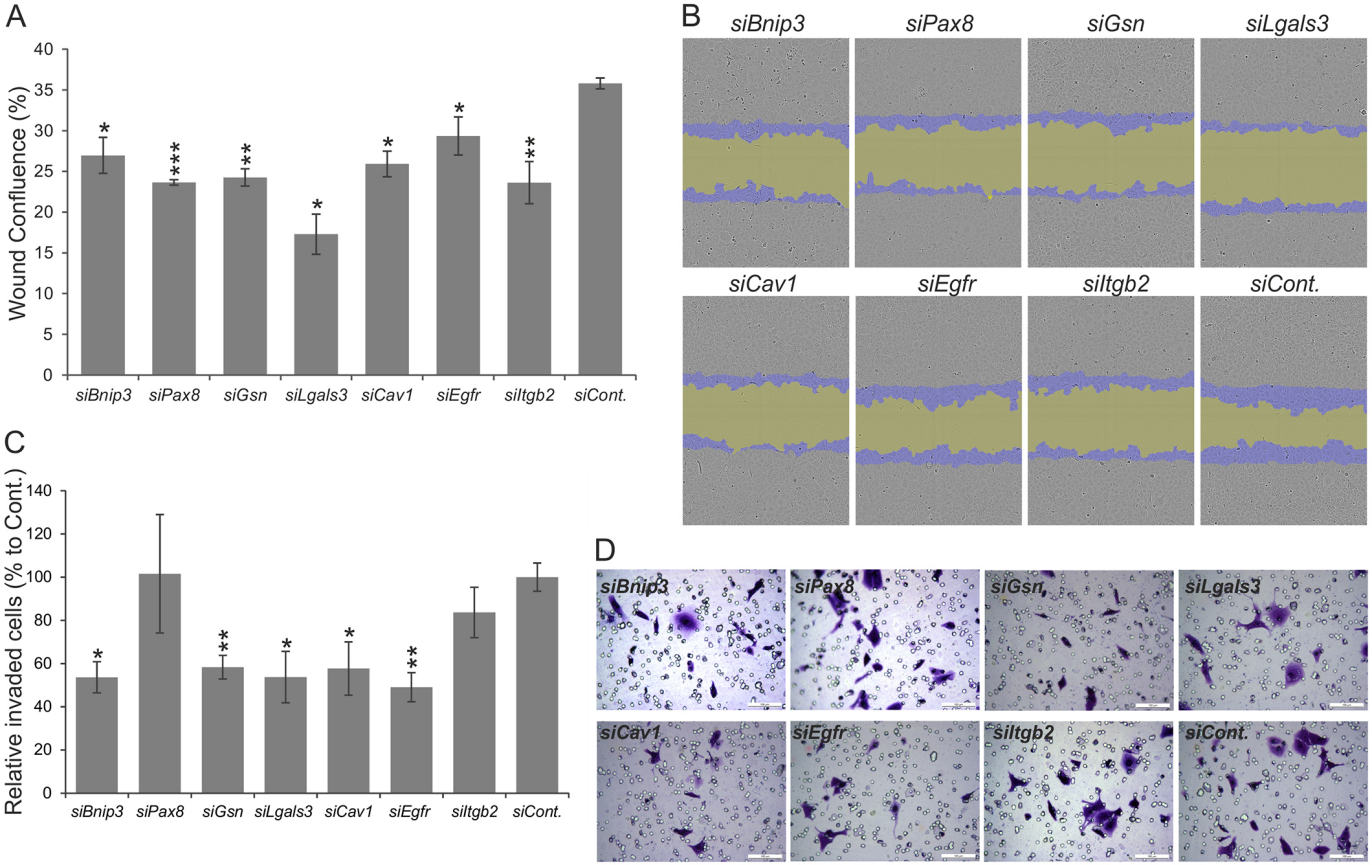
**Effect of siRNA transfection on the migration and invasion of Renca cells.** (A,B) Cell migration analyzed by wound healing assay, as compared with siRNA controls (*siCont.*). Gray, original confluence mask; purple, migrated cells; green, final scratch wound mask. (C,D) Invaded Renca cells analyzed by the Transwell assay, as compared with siRNA controls. The results are presented as means±s.d., and data from three independent experiments are shown. *P<0.05; **P<0.01 and ***P<0.001 compared by *t*-test with control siRNA-transfected Renca cells. Scale bar: 100 μm.

Considering that Renca cells also serve to address mechanisms of metastases to the lung and liver when implanted in mice (Salup et al., 1985), we investigated whether downregulation of the expression of the targeted selected genes would influence the migratory or invasive properties of these cells. For this, we applied siRNA cell transfection with the identified efficient siRNA *in vitro* protocol in 96-well plates. Putative changes in cell migration were monitored by the IncuCyte live-cell imaging system for 24 h after wounding the confluent cell monolayer. The results revealed that downregulation of the selected model genes notably inhibited cell migration in the wound assay (Fig. 4A,B).

Next, we compared the capacity of the siRNA-transfected cells for invasion through a porous filter in a Transwell assay. It turned out that transfection of the selected siRNAs, excluding *Pax8* and *Itgb2*, significantly inhibited cell invasion across the filter compared to the controls (Fig. 4C,D). Taken together, the data indicates that the siRNA-mediated silencing of *Bnip3*, *Cav1*, *Gsn*, *Egfr* and *Lgals3* functions reduces the migratory and invasion capacity of RCC (Renca) cells, whereas silencing of the *Pax8* or *Itgb2* genes deregulates cell migration only.

**Fig. 5.**
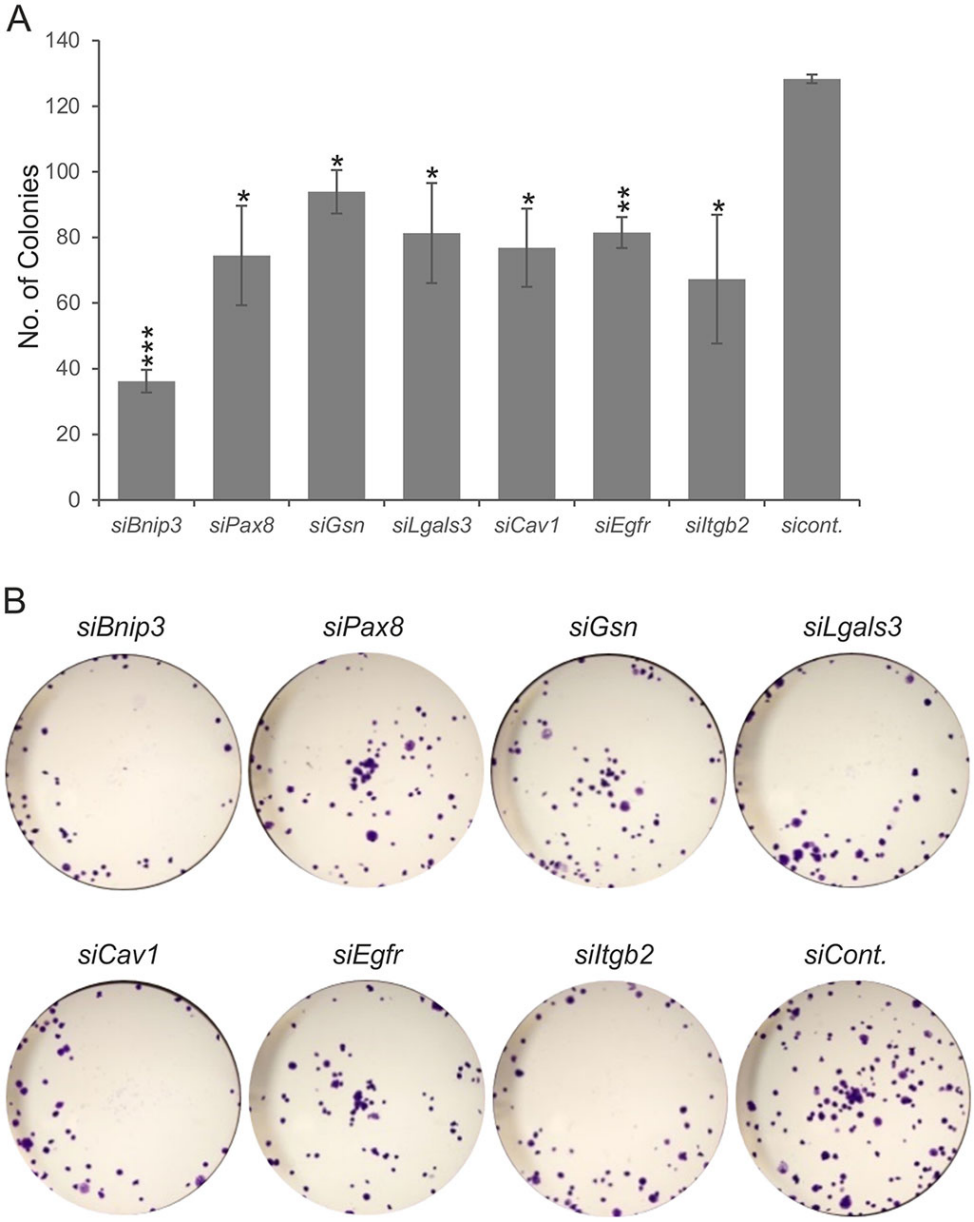
**Effect of siRNA transfection on colony formation by Renca cells.** (A) The number of colonies after siRNA (*si*) transfection; (B) representative photomicrographs. The colony-formation assay was performed by seeding 200 cells/well on a 24-well plate. The number of colonies was counted after 1 week of incubation. Data are presented as means±s.d. Results from three independent experiments are shown. *P<0.05; **P<0.01 and ***P<0.001 compared by *t*-test with control-siRNA (*siCont.*)-transfected Renca cells.

Given the illustrated capacity of the *Bnip3*, *Cav1*, *Gsn*, *Egfr* and *Lgals3* genes to influence activities of the model RCC cells, we continued our siRNA analysis by performing a colony-forming assay. As shown in Fig. 5A and B, the siRNA-transfected cells had a poor capacity to form cell colonies when compared to the controls. The *Bnip3-*specific siRNA turned out to be the most effective, and reduced the amount of colonies by about 70% compared to the controls.

### Silencing of the functional RCC/kidney developmental genes influences EMT/MET markers and phosphorylation of the signal transduction component Akt in the RCC model

Because the embryonic kidney tubulogenesis model serves to study the MET and because the reverse process, EMT, is typical for cancer, we examined whether the identified functional genes have an impact on the EMT and MET processes. Vimentin served as a marker of mesenchymal cells, whereas N-cadherin and E-cadherin were used as epithelial markers for RT-qPCR.

After siRNA silencing, the EMT markers were detected in Renca cells at 48 h. The results showed that introduction of siRNA for *Gsn* to the cells notably downregulated E-cadherin expression (*P*<0.05), but concurrently upregulated expression of the vimentin gene (Fig. S2A). Silencing of the *Bnip3* function upregulated E-cadherin expression, but the mesenchymal marker analysis did not reveal any changes. This was also the case for *Cav1* and mock siRNAs (Fig. S2A).

Because there is evidence that changes in PI3K/Akt pathway signaling can be associated with the RCC phenotype (Cancer Genome Atlas Research Network, 2013), we investigated whether the identified functional siRNAs also have an impact on Akt signaling in cultured Renca cells. We used the degree of Akt phosphorylation as a criterion. However, among the siRNAs used, only *Pax8* siRNA led to enhanced Akt phosphorylation, whereas the *Bnip3*, *Cav1* and *Gsn* siRNAs did not have a significant influence (Fig. S2B). This points towards a different mechanism of action for *Pax8* when compared to all the other genes investigated in our study. To conclude, it appears that the functional consequences of the gene expression inhibition in RCC are unlikely to take place via MET or EMT processes or via different degrees of PI3K/Akt activation.

### siRNA gene-silenced Renca cells in co-culture with embryonic kidney cells as a novel functional *ex vivo* organoid tumorigenesis model

Traditionally, cancer-associated gene functions have been studied either in monolayer cell cultures *in vitro* or more recently by making *in vivo* models via gene editing in the mouse. To be able to conduct more relevant high-throughput functional screens for cancer gene discovery *in vitro*, 3D tumor models that better mimic the natural cellular setting need to be developed.

To develop relevant *ex vivo* diagnostic renal cancer models, we made use of the already described embryonic kidney-tubule induction system in which emergence of a complex kidney tubular network can be achieved with a Wnt-pathway-mediated trigger (Halt and Vainio, 2012). Here, a 24-h induction pulse is enough to trigger tubulogenesis (Saxén, 1987). We recently developed a number of powerful renal tissue engineering capabilities that allow us to generate chimeric organoids and culture them as 3D explant cultures (Junttila et al., 2015; Halt et al., 2016).

**Fig. 6.**
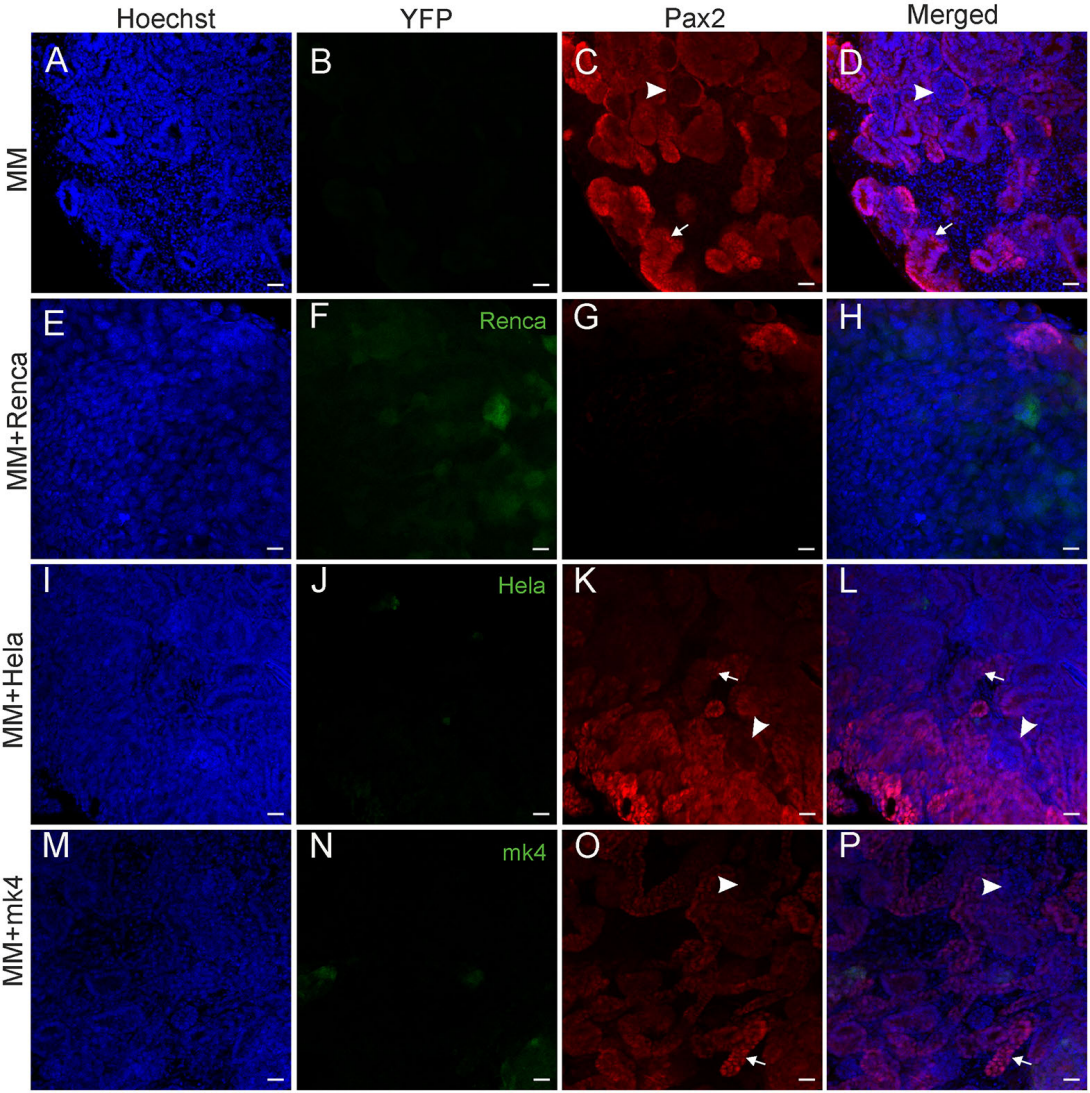
**Disruption of the tubule formation by immortalized cells during nephrogenesis *in vitro*.** After dissociation and reaggregation of embryonic kidney, MM cells were mixed at a ratio of 50:1 with either Renca-YFP, HeLa-YFP or mK4-YFP cells. (A-D) The Pax2+ tubular epithelial structures (in red) and glomeruli (arrowheads) were formed from dissociated and reaggregated embryonic kidney mesenchymal progenitor cells. (E-H) Mixing of Renca-YFP cells to MM strongly impaired tubular-structure formation, whereas addition of HeLa-YFP (I-L) or mK4-YFP (M-P) cells had a much milder effect on the nephrogenesis *in vitro*. 3D cultures were maintained for 4 days. Blue, nuclear stain (Hoechst); green, YFP; red, Pax2 immunostaining. Arrows indicate tubular epithelial structures. Scale bars: 20 µm.

The MM cells were dissociated by collagenase treatment, pelleted by centrifugation and the nephrogenesis program was induced with a Wnt signaling inducer (Junttila et al., 2015; Halt et al., 2016). The reaggregated MM cells were placed on a nuclepore filter and cultured for 4 days. The developmental activation led, as expected, to the appearance of epithelial tubular structures formed by the Pax2-positive cells, depicting successful tubule induction (Bouchard et al., 2002) (Fig. 6A-D).

In order to test how the addition of immortalized cells would influence the normal nephrogenesis process in these 3D cultures, we mixed mesenchyme cells at a 50:1 ratio with renal-carcinoma-derived Renca-YFP cells, cervical-cancer-derived HeLa-YFP cells and mK4-YPF cells, derived from induced MM undergoing epithelial conversion (Valerius et al., 2002).

Interestingly, we found that various stable cell lines behaved markedly differently in the chimera co-culture with the mesenchymal cells. The addition of mouse Renca cells disturbs the differentiation of the 3D kidney culture (Fig. 6E-H) much more strongly than the chimerism with human HeLa cells (Fig. 6I-L) or mouse embryonic-kidney-derived immortalized mK4 cells (Fig. 6M-P). The Renca cells distributed rather evenly within the reconstituted embryonic kidney tissue, whereas HeLa and mK4 grew as small clusters inside the differentiated MM. Therefore, we concluded that RCC-derived cells must be suitable for use in 3D co-cultures to study whether the downregulation of certain genes in RCC could lead to differentiation improvements in the kidney organoids.

**Fig. 7.**
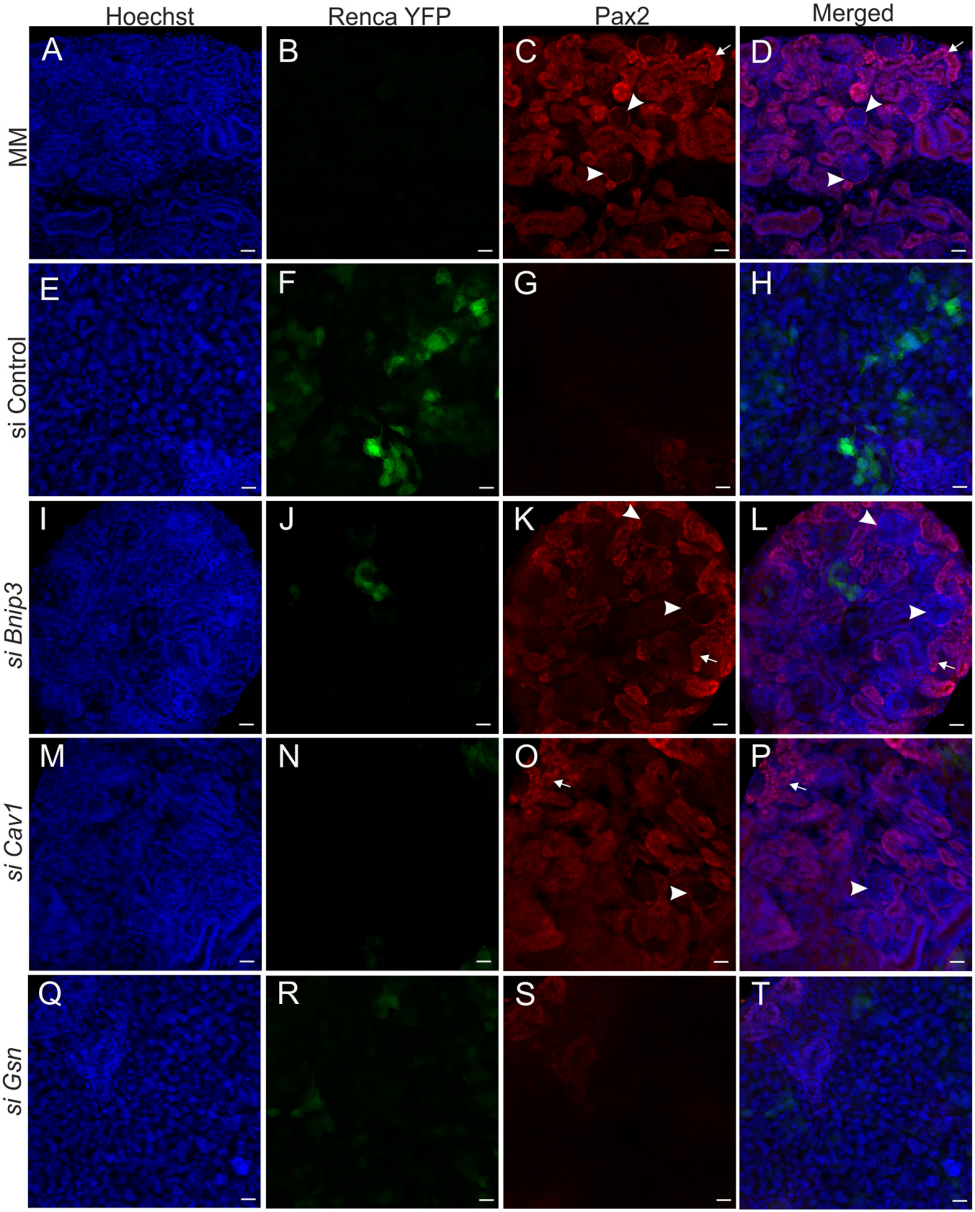
**Nephrogenesis *in vitro* can be rescued in 3D co-cultures by siRNA treatment of Renca cells.** (A-D) The Pax2+ tubular epithelial structures (in red) and glomeruli (arrowheads) were produced by dissociated and reaggregated embryonic kidney mesenchymal progenitor cells. (E-H) Few Pax2+ tubular epithelial structures were formed by MM after mixing with the control-siRNA-treated Renca cells. The Pax2+ tubular epithelial structures and glomeruli formation was again well visible after addition of Renca cells treated with *Bnip3* siRNA (I-L) and *Cav1* siRNA (M-P). These structures also partially formed if the Renca cells were treated by with *Gsn* siRNA (Q-T). 3D cultures were maintained for 4 days. Blue, nuclear stain (Hoechst); green, YFP; red, Pax2 immunostaining. Arrows indicate tubular epithelial structures. Scale bars: 20 µm.

**Fig. 8.**
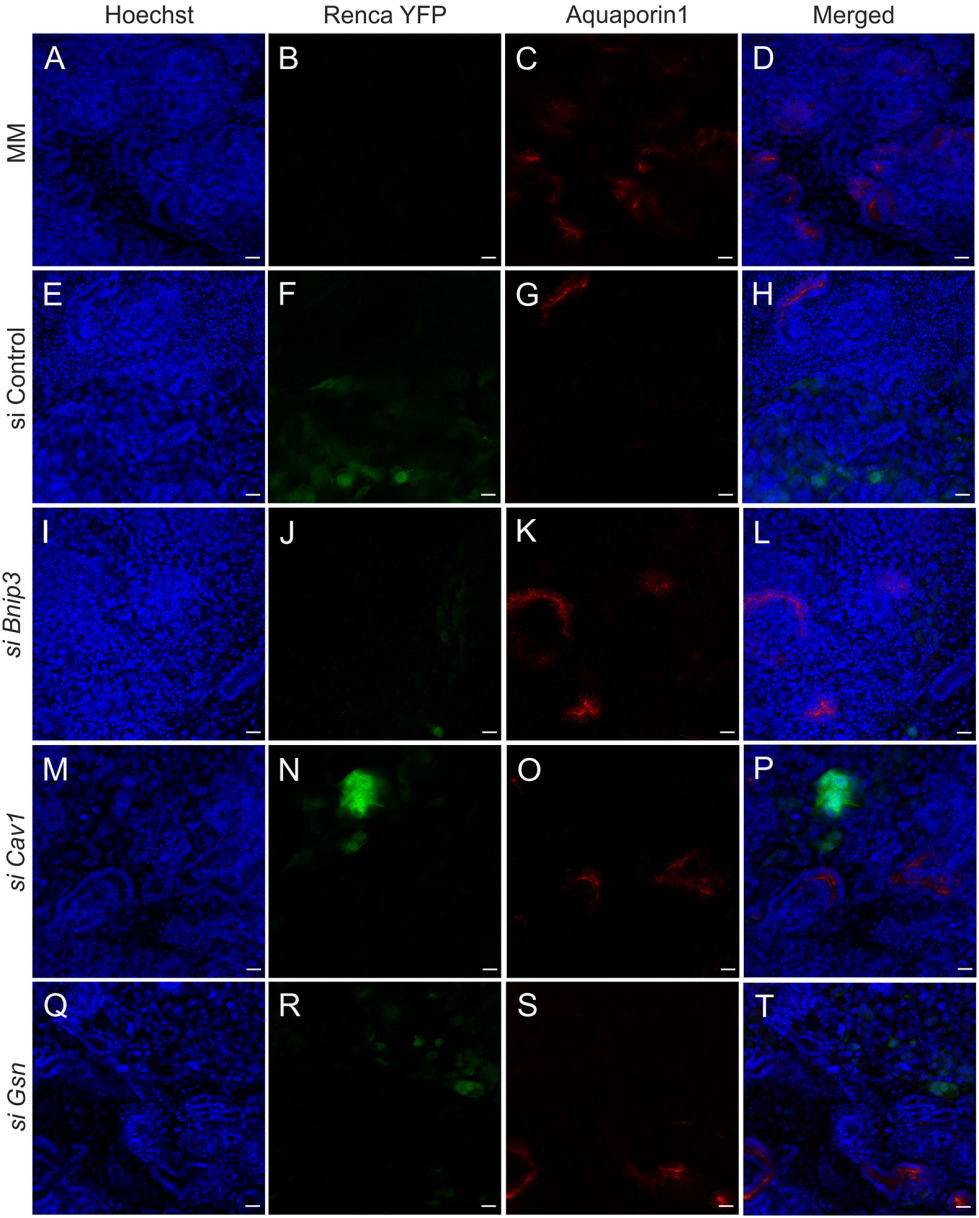
**Formation of proximal tubes can be rescued in 3D co-cultures by siRNA treatment of Renca cells.** (A-D) The proximal tubes (Aq1 staining in red) were well formed in the dissociated and reaggregated MM pellet *in vitro*. Few Aq1+ structures were formed after mixing with the control-siRNA-treated Renca cells (E-H). The proximal tubes were well visible after addition of the *Bnip3* (I-L) and *Cav1* (M-P)-siRNA-treated Renca cells. *Gsn* siRNA treatment also partially rescued tube formation (Q-T). 3D cultures were maintained for 4 days. Blue, nuclear stain (Hoechst); green, YFP; red, Aq1 immunostaining. Scale bars: 20 µm.

Our next goal was to investigate whether inhibition of the genes that showed the most consistent results in our *in vitro* assays (*Bnip3*, *Cav1* and *Gsn*) would also have an effect in 3D co-cultures. Similarly to the untreated Renca-YFP cells, when mock-siRNA-transfected Renca cells constitutively expressing YFP were cultured as a chimera with normal MM cells (1:50), the Renca cells notably inhibited tubulogenesis (Fig. 7A-D versus E-H and Fig. 8A-D versus E-H).

However, when Renca-YFP cells that have been transfected with *Bnip3-* or *Cav1*-specific siRNA were cultured as chimera organoids with wild-type MM cells, multiple Pax2-positive tubular structures and proximal tubes [aquaporin-1 (Aq1)+] were observed (Fig. 7I-P and Fig. 8I-P). The *Gsn*-siRNA-treated Renca cells in the reaggregated pellet disrupted the tubule structure formation, but to a lesser extent than the control siRNA-treated Renca cells (Fig. 7Q-T versus A-D and Fig. 8Q-T versus A-D).

We next aimed to investigate whether increased cell death upon Renca treatment with different siRNAs was the only reason for better formation of Pax2-positive tubular structures in chimeric organoids. Because the application of *Bnip3* siRNA to Renca cells increased cell death the most (by about 250%), we mixed *Bnip3*-siRNA-treated Renca cells with MM at a higher ratio (1:20). The results showed that many renal tubules were still formed even at this ratio, showing that, in addition to apoptosis, some other changes in Renca cells upon siRNA treatment contribute to their growth in co-culture with MM (Fig. S3).

In general, our data indicate that downregulation of the expression of certain genes in RCC (Renca) cells co-cultured with MM could rescue tubule formation, which was disrupted by wild-type Renca cells, showing parallels with the *in vitro* results described earlier (Figs 3–5). In summary, the results reveal that embryonic kidney nephron progenitor cells that are first dissociated and then later used to reconstitute the nephrogenic potential provide a novel and complex cancer gene discovery model.

## DISCUSSION

Despite the fact that some of the genetic changes underlying ccRCC have been described before (Cancer Genome Atlas Research Network, 2013), there are still no clinically applicable prognostic markers for this disease (Parkinson et al., 2014). Better understanding of the molecular pathogenesis of RCC elucidates that carcinogenesis and nephrogenesis share certain properties (Aiello and Stanger, 2016). This consideration is based on the fact that some of the gene expression signatures observed in kidney development are shared with cancer cells (Kim and Orkin, 2011).

Given the depicted facts, we first aimed to find out whether a comparison of the expression profiles during nephrogenesis and kidney cancer development may help identify novel renal-cancer-inducing genes. Next, we planned to address the putative roles of the identified genes as functional players in cell growth control using not only classical *in vitro* assays but also a novel *ex vivo* assay system based on our recent success in dissociating and reconstituting the embryonic kidney from founder cells (Junttila et al., 2015; Halt et al., 2016). More specifically, we aimed to establish novel and relevant organoids representing a chimera between embryonic kidney cells that normally assemble the nephron and RCC cells. The data indicates that our development of a novel complex assay system was successful. The generated setup allows co-culture of the RCC cells with the host embryonic kidney cells to mimic RCC tumorigenesis in an *ex vivo* setting suitable for RCC functional studies.

Given the similarities between developmental processes and the processes involved in cancer, including for example extensive cell proliferation and changes in cell differentiation, we used the classic mammalian kidney-tubule induction model in the hope of identifying candidate developmental control genes. The candidate genes were then compared to genes highly expressed in a human RCC cohort.

The microarray-mediated analysis led to the identification of multiple genes with similar expression patterns in developing embryonic kidney cells and in renal carcinogenesis. Ingenuity pathway analysis found that products of these genes are involved in multiple intracellular processes. Interestingly, pathways found by analyzing only genes that are changed in both processes simultaneously gave different results compared to analyzing the cancer dataset and developmental dataset separately (Fig. 1A and Table S3). Taking into account the *P*-values and percentages of involved genes from Ingenuity analysis software as well as literature data outlined below, we selected *Cav1* and other members of caveolar-related pathway to study in further detail.

Caveolae represent invaginations of the plasma membrane of around 50-100 nm, connected to endocytosis, lipid regulation and signal transduction (Parton and del Pozo, 2013). Loss of caveolae, as a result of mutations or gene expression changes in caveolins or cavins, results in the development of various diseases, including cancer (Martinez-Outschoorn et al., 2015). Caveolae interact with membrane-lipid rafts and the galectin lattice controlling cell signaling both under normal physiological conditions and in cancer (Dumic et al., 2006; Lajoie et al., 2009; Shankar et al., 2015). Caveolins, a major constituent of the caveolae, have been shown to be important for the regulation of many signaling cascades involved in cancer development.

In the past 20 years, dysregulated *Cav1* expression has consistently been detected in various cancers (Martinez-Outschoorn et al., 2015). Still, there is no universal pattern linking *Cav1* expression in cancer cells and patient outcomes: high *Cav1* levels are associated with poor prognosis and aggressive disease in melanoma, breast, prostate and lung cancer, whereas, at the same time, high *Cav1* expression is correlated with good clinical outcomes in head and neck cancer and extrahepatic biliary carcinoma cells (Martinez-Outschoorn et al., 2015). In renal cancer, *Cav1* has been shown to be a tumor progression factor, but in general its involvement in kidney tumorigenesis has been investigated relatively little (Campbell et al., 2013; Steffens et al., 2011; Waalkes et al., 2011).

Caveolins, including caveolin-1, are expressed in mouse embryos in a tissue-specific manner starting from E6.5 (Sohn et al., 2016). Caveolin-1 is also involved in the regulation of signaling pathways critical for nephrogenesis, including the Wnt pathways (Parton and Simons, 2007), and is known to be connected to Wnt/β-catenin signaling in podocytes (Jing et al., 2015). Still, no studies that explain the function of caveolins in kidney organogenesis, or even in embryogenesis in general, have been reported to date (Sohn et al., 2016).

Owing to the presence of a scaffolding domain, caveolins interact directly with signaling molecules, including cytokine receptors, and suppress the activation of EGFR upon EGF stimulation (Agelaki et al., 2009; Martinez-Outschoorn et al., 2015; Lajoie et al., 2009; Park et al., 2001). On the other hand, galectin-glycoprotein lattices can sequester EGFRs, protecting them from interacting with caveolin-1 scaffolds, and hence promoting GF signaling and tumor growth (Lajoie et al., 2007).

One potential role for galectins and caveolins might be to regulate crosstalk between integrin and various signaling molecules (del Pozo et al., 2005; Lajoie et al., 2009). Electron microscopy has revealed complex interactions between caveolae and cytoskeleton components such as actin filaments (Parton and del Pozo, 2013). Gelsolin, an actin-binding protein with a well-established role in actin organization (McGough et al., 2003), has been recently described for the first time as a caveolar raft-associated molecule (Chillà et al., 2013). Among the downstream targets of caveolin-1 are proteins involved in autophagy induction, including *Bnip3* (Martinez-Outschoorn et al., 2015). We found here that the developmentally regulated genes *Cav1*, *Egfr*, *Bnip3*, *Gsn*, *Itgb2* and *Lgals3* are all activated in human ccRCC.

When comparing the effects of gene downregulation by siRNA on cell viability, we observed that only *Cav1-*, *Gsn-* and *Bnip3*-siRNA-treated cells exhibited lower cell proliferation than the controls. The strongest reduction in colony number in the colony-forming assay was observed after transfection of cells with *Bnip3*-specific siRNA. Moreover, all the siRNAs tested, except for *Egfr* siRNA, induced cell death, although they did so to different extents. Again, the most prominent effects were seen for *Bnip3* and *Gsn*. In general terms, these results suggest that higher cell death rather than a decrease in cell proliferation explains the reduction in Renca cell numbers after transfection with the *Bnip3-* and *Gsn-*specific siRNAs.

We also found here that downregulation of all the selected genes reduced the motility of Renca cells in the wound healing assay. In addition, the Transwell assay showed that *Cav1*, *Gsn*, *Bnip3*, *Lgals3* and *Egfr*, but not *Pax8* or *Itgb2*, have an effect on Renca cell invasion. Given this fact, we may conclude that a number of developmental genes related to caveolar signaling promote kidney tumorigenesis in a coordinated manner. Indeed, our data demonstrated that downregulation of each of the selected genes had an effect on at least some of the cell behavioral features commonly associated with carcinogenesis. Only three genes – *Bnip3*, *Cav1* and *Gsn* – however, were found to be effective in all the assays tested.

Our results are generally in line with previously reported data. *Cav1* gene expression has been observed to coordinate with *Lgals3* in several cancers, and their encoded proteins together have been seen to regulate the downstream pathway signaling (Goetz et al., 2008; Lajoie et al., 2007; Shankar et al., 2012). These observations had not been reported for renal cancer before.

*In vitro* downregulation of *Gsn* in breast cancer MDA-MB 231 and in prostate cancer PC-3 cell lines reduced the cells' invasive and motile properties, as well as cell aggregation (Van den Abbeele et al., 2007). Indeed, we noted here that downregulation of *Gsn* influenced RCC cell behavior, probably by targeting the process of EMT, which is critical for normal nephrogenesis and, when deregulated, also for tumorigenesis. Depending on the cellular context, expression of *Bnip3* either induces or delays cell death, and overexpression of *Bnip3* has been reported in several types of cancer (Burton et al., 2006). Our data indicate that in renal carcinoma cells, *Bnip3* acts as an anti-apoptotic factor.

Components of the PI3K/Akt pathway belong alongside HIF/VHL to the group of the most frequently altered genes in ccRCC (Guo et al., 2015). The PI3K pathway has recently also been shown to balance self-renewal and differentiation of nephron progenitor cells during kidney development (Lindström et al., 2015). Moreover, there is a link between caveolin and Akt signaling. The activation of the caveolin-1/PI3K/Akt/GSK3β pathway has been found to mediate the cardioprotective effect of epigallocatechin-3-gallate in cardiac cells (Hsieh et al., 2013), but it is also responsible for ammonium-related toxicity in astrocytes (Wang et al., 2017).

We studied whether downregulation of caveolin-related genes influences Akt phosphorylation in Renca cells and found that inhibition of these genes did not lead to significant changes in Akt activation. However, downregulation of *Pax8* resulted in a strong induction of Akt phosphorylation. This may indicate that Pax8 has a different action mechanism from the other genes used in our study.

Next, we developed 3D co-cultures that make it possible to study the cross-interactions between embryonic and transformed cells under conditions in which the expression of certain genes that are relevant both for normal development and carcinogenesis is inhibited. We found that the addition of immortalized cells of different origin reduces kidney tubule formation in the 3D chimera assay. The level of this reduction varies from quite slight for the mK4 embryonal-derived cell line to very severe for the Renca renal-carcinoma-derived cells.

The fact that the RCC cells inhibit normal tubulogenesis is consistent with our earlier finding. We have shown that, for example, Wilms tumor inhibits Wnt4 function and promotes kidney tumorigenesis. Thus, mutation of WT1 maintains progenitor characteristics of kidney cells. This mutation also prevents MET (Murugan et al., 2012). Importantly, in the present study, *Bnip3*, *Gsn* and *Cav1* downregulation in Renca cells with siRNA compounds enabled tubule formation as judged from the induction of *Pax2* and *Aq1* expression in the induced kidney mesenchymal cells. Therefore, the kidney tubules are able to develop when the expression of certain genes was silenced with siRNAs prior to establishment of the organoid chimera.

To conclude, it appears that the downregulation of several embryonic developmental genes by siRNA treatment reduced the malignant behavior of renal cancer cells. In addition to providing a way to identify putatively novel oncogenes, the current work also offers a novel platform to study the capacity of kidney cancer cells to influence normal cell proliferation, differentiation and morphogenesis in a close to *in vivo* setting. Interestingly, this model also provides a way to monitor how normal kidney cells influence cancer growth upon silencing of carcinogenesis-related genes.

The *ex vivo* setup may also be extended to an *in vivo* situation by grafting the reaggregated organoid under the kidney capsule. The proposed techniques make it possible to monitor cancer cells and correlate their behavior to the process of organogenesis. The siRNA-mediated inhibition of nephrogenesis genes in kidney cancer may not only help to identify new putative oncogenes but may also provide new therapeutic opportunities.

## MATERIALS AND METHODS

### Patients and RCC samples

A biobank of human ccRCC biopsies (including a control region in which cells appeared histologically normal) was generated from donors (*n*=16) treated in the Haukeland University Hospital (Bergen, Norway) during 11.2013-08.2014. All the experiments were approved by the regional ethics committee of Western Norway (REC West no. 78/05) and by written consent of the participants.

### Animals

Embryos from wild-type CD1 mice were used to prepare the embryonic kidneys at E11.5 for the *ex vivo* chimera organoid assays. Maintenance of the colonies and collection of the embryos were performed in accordance with the Finnish national legislation, European Convention ETS 123 and EU Directive 86/609/EEC, and were approved by the local ethics committee.

### Renca cell culture

Mouse Renca cells (ATCC^®^ CRL-2947™) and HeLa cells were obtained from the American Type of Culture Collection (ATCC) and had been verified via short tandem-repeat profiling. The mK4 cell line has been described previously (Valerius et al., 2002). The cells were passaged in Dulbecco's modified Eagle's medium (DMEM) supplemented with 10% fetal bovine serum (FBS), 100 U/ml penicillin and 100 µg/ml streptomycin in 5% CO_2_ at 37°C. YFP-Renca, -HeLa and -mK4 cells were generated by transfection with the *pcDNA3.1^+^ YFP* cDNA construct. Cells in which the construct had integrated into the genome were enriched via puromycin-mediated selection and FACS (BD FACSAria™ IIIu). The YFP-expressing cells were used to make the chimeric organoids with embryonic kidney nephron progenitor cells. A panel of siRNAs (Sigma-Aldrich) was used to knock down their respective target mRNAs. The MISSION siRNA (Sigma-Aldrich) was used as a control.

### Transfilter kidney-tubule induction assay

Embryonic kidneys were dissected from CD1 embryos at E11.5 and incubated in 1.125% pacreatin-2.25% trypsin for 30-40 s. The ureteric bud was separated from the nephron-progenitor/stem-cell-containing MM in media supplemented with 10% FBS and antibiotics (Saxén, 1987). The MM was treated with 40 µl of 2 mg/ml collagenase III in 280 µl of physiological buffer at 37°C for 10 min to obtain a single-cell suspension. The MM cells were washed twice with cell culture medium, placed on Nuclepore polycarbonate membrane (Whatman, pore size 1.0 µm), and a piece of embryonic dorsal spinal cord (E11.5) was glued onto the other filter side as a robust tubulogenesis inducer. The conjugate was cultured in 37°C, at 5% CO_2_, for 96 h, which is sufficient to induce and advance nephrogenesis (Saxén, 1987), snap frozen in liquid nitrogen, and stored at −80°C until used for RNA purification. Collectively, 40 freshly prepared control and induced MM were processed for the oligonucleotide gene-chip analysis. The analyses were conducted in triplicates.

### Microarrays and statistical analysis

Total RNA (200 ng) that had been extracted from control and induced MM was subjected to Affymetrix Mouse Genome 430 2.0 array analysis. The Affymetrix cell-derived data files were processed with the R Bioconductor LIMMA package (Ritchie et al., 2015). The robust multichip average (RMA) (Irizarry et al., 2003) tool served to adjust the signal-to-noise ratio and was also normalized with Quantile by median polished probe-set summarization with the perfect data match. The data values with log_2_>6.64 scores in at least three of the six analyzed gene-chip samples were considered a relevant change. The expression was counted as significant when its fold change was >2.0 with a *P*-value of <0.05 adjusted with the Benjamini–Hochberg multiple testing correction tools.

For the patient-derived RCC and control samples, two biopsies from each patient were taken. The first one was processed for histopathological inspection and ccRCC grading. The other sample was taken from the same tissue but from a region that appeared histologically normal. Control and RCC samples were stored until subjected to RNA purification for the generation of a gene library. The genes that depicted a fold change of >2.0 with *P*<0.05 were considered to be differentially expressed (Eikrem et al., 2016).

The Ensemble IDs of the genes that turned out to be differentially expressed in the induced MM cultured for 96 h and the human ccRCC samples were analyzed with the Ingenuity Knowledge Base canonical pathway collection tool (www.ingenuity.com, version 23814503). The significance of the genes in the illustrated signal transduction pathways was evaluated first. The data was then presented as the *P*-values corrected with multiple testing via the Benjamini–Hochberg algorithm. Only data that depicted an adjusted *P*-value of <0.05 are reported. The Venn diagram (Fig. 1A) was generated using an online tool (www.pangloss.com/seidel/Protocols/venn.cgi).

Groupwise comparison with linear model for microarray data from GSE53757 (Smyth, 2005) was performed in Geo2R (www.ncbi.nlm.nih.gov/geo/geo2r/). *P*-value adjustment is reported as Benjamini–Hochberg false discovery rate.

### Analysis of cell proliferation and cell death

The degree of proliferation of the Renca cells was estimated with the IncuCyte ZOOM (Essen BioScience Inc.) live-cell imaging system. The siRNAs (50 nM) were transfected to the Renca cells by Lipofectamine RNAiMAX (Thermo Fisher Scientific). Transfected cells were plated and cultured for 2 days, after which the cells were detached by trypsinization, counted and plated into the 96-well plates at a density of 3000 cells/well. The degree of cell confluence in the cultures was monitored every 2 h for 4 days starting 2 h after setting the cultures. For the cell death measurements, 1 µM SYTOX^®^ Green nucleic acid stain (Life Technologies) was added to the cells after seeding into 96-well plates. The putative siRNA-induced cytotoxicity was assayed by comparing the confluence metrics of the total cells and the dead cells using the IncuCyte integrated software.

### Wound-healing-induced cell migration

The putative changes in cell migration caused by siRNA were analyzed by an *in vitro* wound-healing assay using the IncuCyte ZOOM system. The RCC cells were transfected with 50 nM siRNAs for 2 days in culture. After trypsinization and washing with PBS, the cells were plated into Essen ImageLock 96-well plates (40,000 cells/well) and cultured for 20 h to reach 90-100% confluence. Then, 0.4 µg/ml mitomycin C (Sigma) was added and incubated for 4 h to inhibit cell proliferation. The cells were washed and the wounds were generated with the IncuCyte Wound Maker (Essen BioScience Inc.). After a 24 h migration time, the wounds were photographed and analyzed with the software tool included in the IncuCyte platform. Wound confluence (in %) was taken as a measure of cell motility.

### Estimation of cell invasion potential

The invasion capacity of the Renca cells was evaluated by using the Transwell filter assay (6.5 mm diameter, 8.0 µm pores; Corning Costar) as described (Zhou et al., 2013). The Renca cells were transfected with 50 nM siRNAs for 2 days in culture. After trypsinization, 20,000 cells were plated into the upper Transwell chamber. After a 24 h culture period, the culture medium was aspirated. The wells were washed twice with PBS and the cells on the plated filter side were removed. Cells that had already migrated across the filter were fixed with 3.7% formaldehyde, stained with 0.4% crystal violet and counted.

### Colony-forming assay

To study the putative siRNA-mediated cell growth inhibition, Renca cells were transfected with a Lipofectamine siRNA (50 nM) cocktail and cultured for 2 days. The cells were trypsinized, counted, and 200 cells/well were seeded and cultured for 7 days. The formed cellular colonies were fixed with 4% paraformaldehyde (PFA) for 15 min, stained with 0.4% crystal violet. The number of colonies containing more than 50 cells each was counted. Triplicates containing 20-150 colonies/well were counted for each treatment.

### Western blotting

Total proteins (30-50 μg per sample) were extracted from the siRNA-transfected cells. The proteins were separated by gel electrophoresis (10% polyacrylamide gel) and transferred to a nitrocellulose membrane for immunoblotting by using routine procedures. Anti-Akt (Cell Signaling #4691, 1:1000), anti-phospho-Akt (Ser473, Cell Signaling #4060, 1:1000), anti-caveolin-1 (Abcam, ab2910, 1:1000), anti-gelsolin (ThermoFisher, PA5-27350, 1:1000) and anti-α-tubulin (Sigma-Aldrich #T6074, 1:5000) primary antibodies were incubated overnight at 4°C with the membranes, and washed several times in PBST buffer. The respective secondary peroxidase-conjugated IgG antibodies (Invitrogen) were then applied to the membranes. The LumiGLO Peroxidase chemiluminescence kit (Cell Signaling) was used to visualize the bound antibodies.

### RNA isolation and RT-qPCR

Total RNA was extracted from the cells 2 days after siRNA transfection with the RNeasy mini kit (Qiagen). The cDNA was synthesized from 1 µg of total RNA with the First Strand cDNA Synthesis Kit (Thermo Fisher Scientific), and 2 µl of the cDNA in 1:10 dilution was subjected to the RT-qPCR reaction. The Brilliant II SYBR^®^ Green QPCR Master Mix (Agilent Technologies) was used according to the manufacturer's instructions.

The forward and reverse primers for the RT-qPCR were: 5′-CAGTGAGCTTCCCGTTCAG-3′ and 5′-AGAACATCATCCCTGCATCC-3′ (*GAPDH*), 5′-GCTTGGGTTGAAGACAGGAG-3′ and 5′-GTGAAGGCTTGAGCACAACA-3′ (*E-cadherin*), 5′-CCATCCTGACAGACCCCAAC-3′ and 5′-ACTGAGGTGGGTGCTGAATG-3′ (*N-cadherin*), 5′-TCCAGAGAGAGGAAGCCGAA-3′ and 5′-AAGGTCAAGACGTGCCAGAG-3′ (*vimentin*). The RT-qPCR reactions were done in the Mx3005P qRT-PCR System machine (Agilent Technologies). The *GAPDH* probe served as a control to normalize the data. The experiments were repeated at least three times.

### Generation of the chimeras

The dissociation and reaggregation assay of the MM has been described (Junttila et al., 2015). The separated mouse embryonic MM (E11.5) was mechanically dissociated into a single-cell suspension after incubation with 9400 U/ml collagenase type IV for 15 min. Chimeric organoids were assembled by mixing the MM cells and either Renca-YFP, HeLa-YFP or mK4-YFP cells at a 50:1 ratio. In the *Bnip3*-siRNA-treated condition, we also tried a 1:20 ratio mixture with MM. Organoids were then reaggregated in the presence of the nephrogenesis inducer bromoindirubin-3-oxime (BIO) for 20 h in DMEM with 4.5 g/l glucose and 10% FBS at 37°C.

The Renca-YFP cells were treated with siRNA twice: 2 days before and 3 h before co-culture onset. After removal of BIO at 24 h after the start of reaggregation, the chimeric organoids were subcultured for 4 days, fixed with 4% PFA and stained with anti-Pax2 (Abcam ab79389) and anti-Aq1 (Cell Applications.INC CA0648) antibodies as reported (Itäranta et al., 2009).

## Supplementary Material

Supplementary information
