# Supplementary material for: Renal carcinoma/kidney progenitor cell chimera organoid as a novel tumorigenesis gene discovery model
Source: Dis Model Mech. 2017 Dec 1;10(12):1503–15. doi: 10.1242/dmm.028332 (PMC5769601; doi:10.1242/dmm.028332)
Supplement: Supplementary information [file dmm-10-028332-s1.pdf]

Supplementary Figure 1

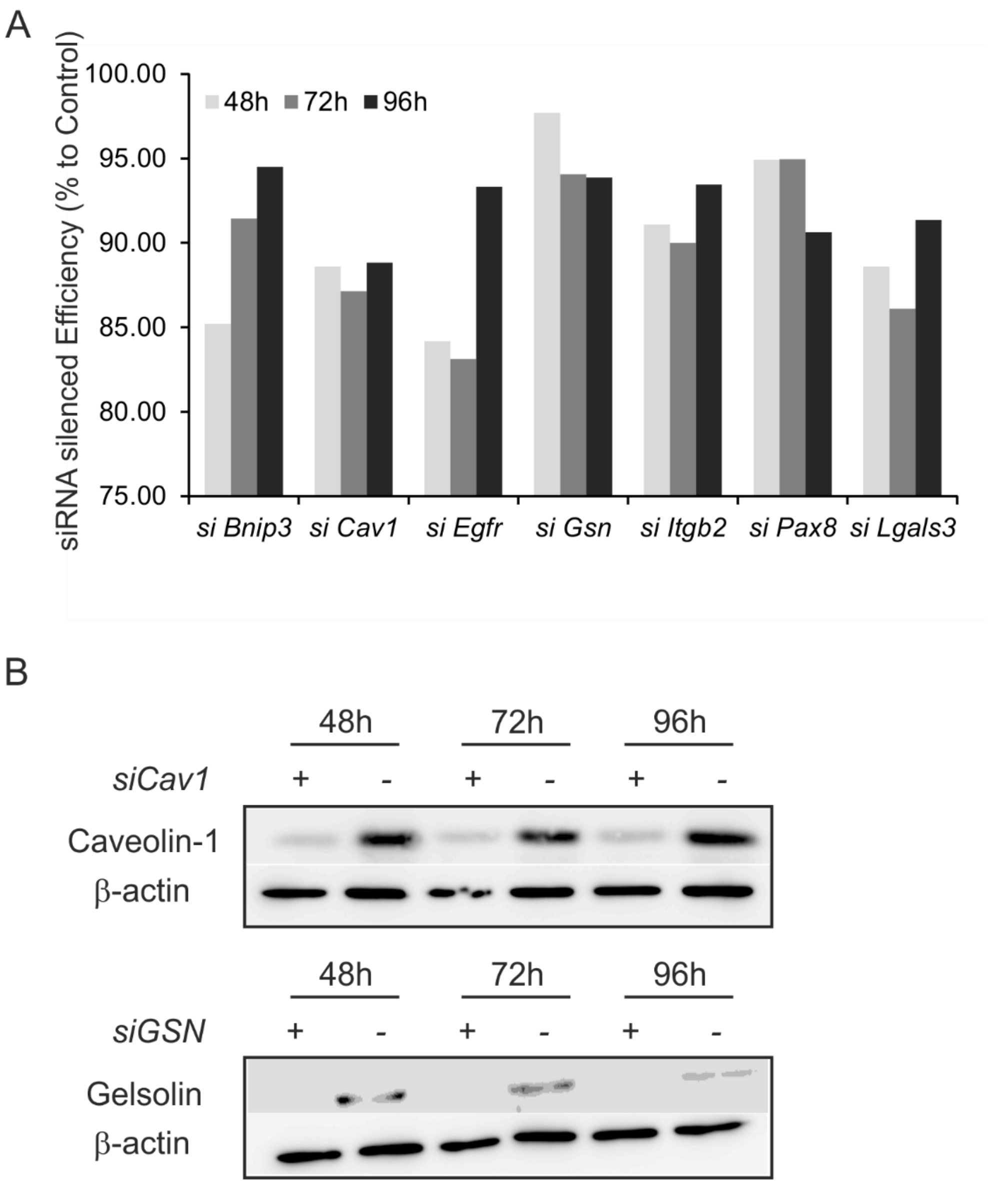

Supplementary Figure 1 Gene expression and protein profiles in siRNA-transfected Renca cells. (A) The mRNA expression level was determined by qRT-PCR. The results are expressed as relative mRNA levels in cells treated with siRNAs for 48h, 72h and 96h. The results obtained with the control siRNA were given the value 1. (B) Western blot analysis of Gsn and Cav1 levels in siRNA treated Renca cells.

Disease Models & Mechanisms • Supplementary information

Supplementary Figure 2

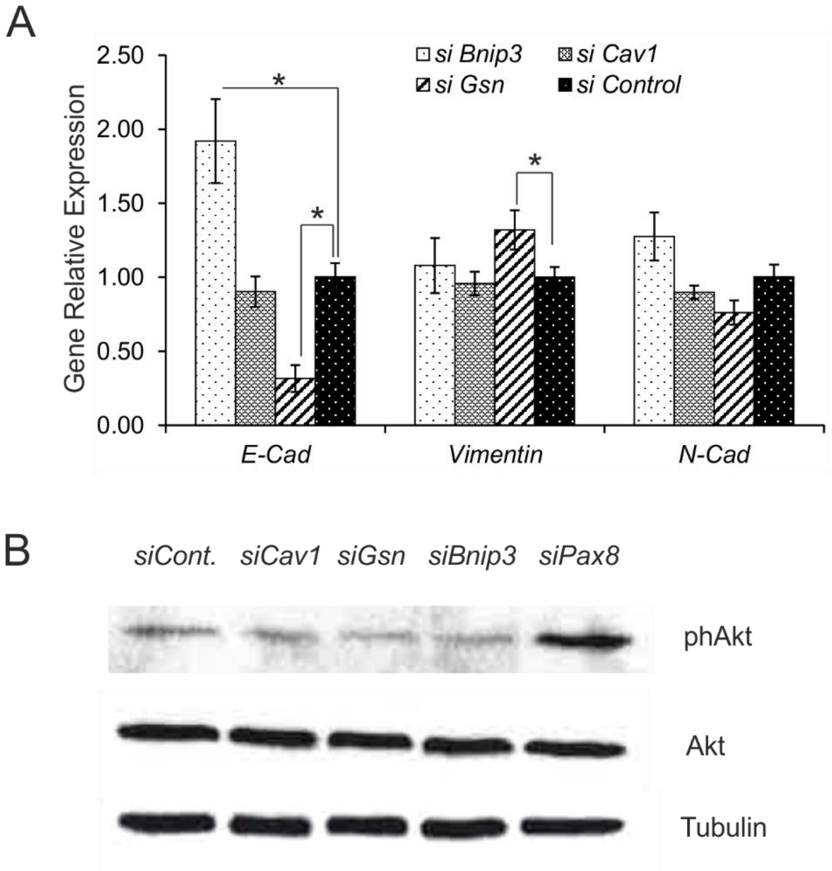

Supplementary Figure 2 Analysis of EMT and Akt pathways in siRNA silenced Renca cells. (A) EMT marker gene expression measured by qRT-PCR. Data are presented as means ± SD, and results from three independent experiments are shown. *p<0.05 compared by t-test with control siRNA-transfected Renca cells. (B) Western blot analysis of phosphorylated Akt, total Akt and alpha-tubulin levels in Renca cells treated with siRNAs.

Disease Models & Mechanisms • Supplementary information

Supplementary Figure 3

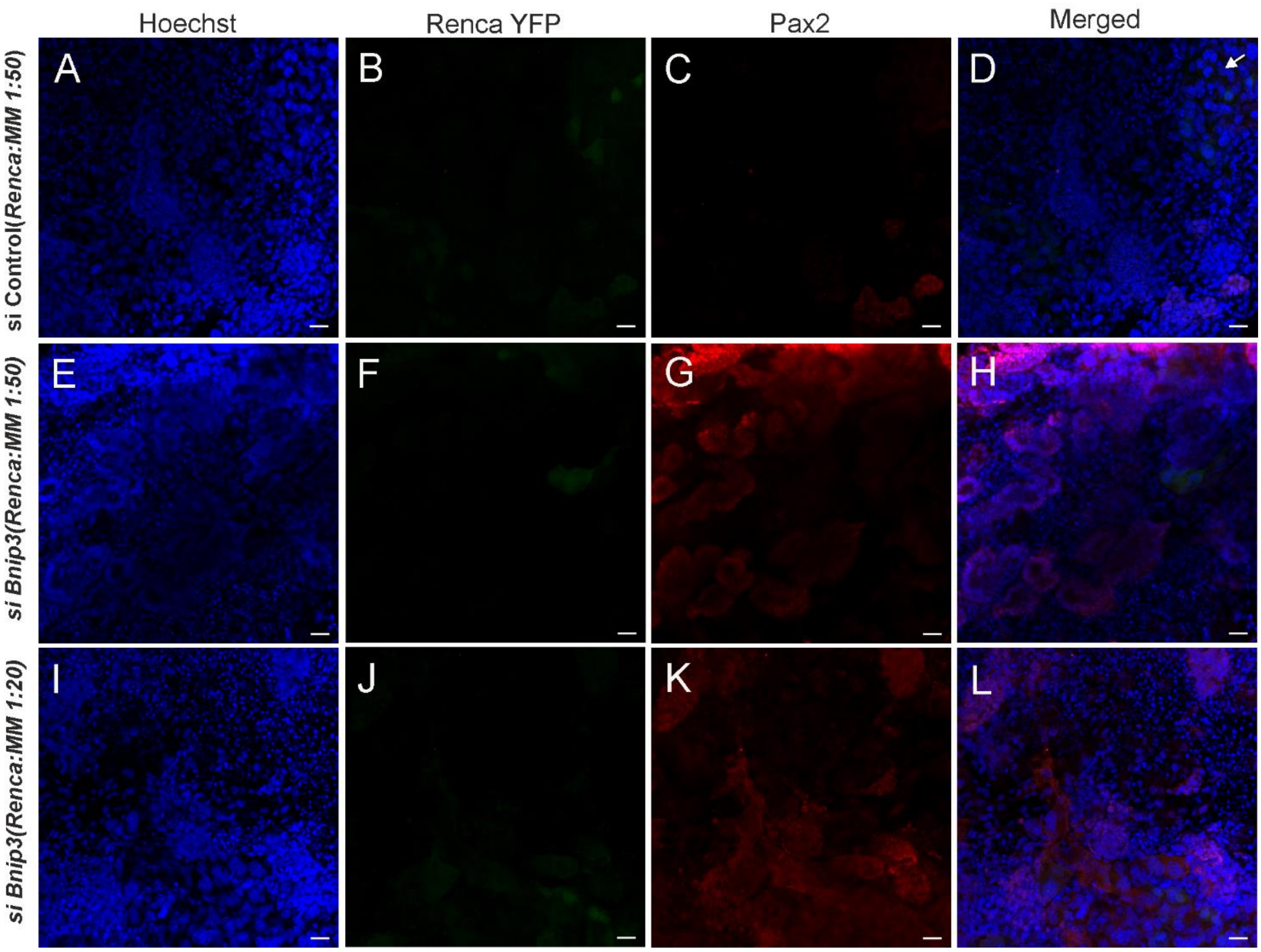

Supplementary Figure 3. Different ratios of *siBnip3* treated Renca to MM cells in the co-culture organoids. (A-D) Formation of Pax2+ tubular epithelial structures was disrupted by the siControl treated Renca cells. (M-P) The Pax2+ tubular epithelial structures were well formed in the organoids with 1:20 ratio (I-L) as well as with the 1:50 ratio of cancer to normal cells (E-H). 3D cultures were maintained for 4 days. Blue - nuclear stain (Hoechst); green – YFP; red – Pax2 immunostaining. Bar: 20µm.

Disease Models & Mechanisms • Supplementary information

Supplementary Table 1 Differentially expressed genes in the induced MM.

Click here to Download Table S1

Supplementary Table 2 Genes differentially expressed in both induced MM and ccRCC.

Click here to Download Table S2

Supplementary Table 3 VENN diagram and pathway lists.

Click here to Download Table S3

Supplementary Table 4 Groupwise comparison with linear model for microarray data from Gene Expression Omnibus database GSE53757.

Supplementary Table 4

Gene.symb	Gene.title	ID	adj.P.Val	P.Value	t	B	logFC (TU/NO)
BNIP3	BCL2/aden	201849_at	4.54E-20	2.80E-21	11.57	37.62	1.14
BNIP3	BCL2/aden	201848_s_	2.29E-15	2.34E-16	9.52	26.35	1.10
GSN	gelsolin	214040_s_	7.04E-17	6.21E-18	10.18	29.96	2.11
GSN	gelsolin	200696_s_	2.01E-15	2.04E-16	9.54	26.49	0.93
GSN	gelsolin	234431_at	3.19E-02	1.76E-02	2.41	-4.76	0.36
GSN	gelsolin	227957_at	6.58E-02	3.95E-02	2.08	-5.46	0.54
GSN	gelsolin	234240_at	1.41E-01	9.39E-02	1.69	-6.18	0.46
GSN	gelsolin	227958_s_	5.95E-01	5.13E-01	-0.66	-7.38	-0.16
CAV1	caveolin 1,	212097_at	3.11E-46	2.96E-49	24.87	101.75	3.05
CAV1	caveolin 1,	203065_s_	1.17E-38	5.11E-41	20.47	82.91	2.75
EGFR	epidermal g	224999_at	1.62E-35	1.16E-37	18.82	75.22	1.95
EGFR	epidermal g	201983_s_	1.76E-32	1.99E-34	17.30	67.80	2.03
EGFR	epidermal g	210984_x_	3.90E-30	6.36E-32	16.15	62.05	3.23
EGFR	epidermal g	201984_s_	9.50E-23	4.27E-24	12.75	44.08	1.82
EGFR	epidermal g	211607_x_	3.94E-21	2.16E-22	12.04	40.18	2.72
EGFR	epidermal g	1565483_a	1.55E-04	5.40E-05	4.19	0.63	0.99
EGFR	epidermal g	1565484_x	1.43E-01	9.51E-02	1.68	-6.19	0.33
EGFR	epidermal g	211551_at	3.86E-01	3.04E-01	-1.03	-7.07	-0.13
ITGB2	integrin, be	1555349_a	5.51E-31	7.83E-33	16.56	64.14	2.32
ITGB2	integrin, be	202803_s_	4.25E-30	6.95E-32	16.14	61.96	2.39
ITGB2	integrin, be	236988_x_	2.45E-08	5.18E-09	6.30	9.62	1.74
LGALS3	lectin, gala	208949_s_	4.63E-18	3.58E-19	10.69	32.79	0.93
LGALS3	lectin, gala	1557197_a	3.15E-04	1.15E-04	3.99	-0.09	0.62
PAX8	paired box	121_at	1.63E-34	1.34E-36	-18.31	72.78	-1.33
PAX8	paired box	209552_at	2.47E-26	7.05E-28	-14.37	52.76	-1.35
PAX8	paired box	221990_at	1.38E-20	8.07E-22	-11.80	38.86	-1.05
PAX8	paired box	207924_x_	1.22E-16	1.10E-17	-10.08	29.39	-1.12
PAX8	paired box	207923_x_	7.03E-16	6.83E-17	-9.74	27.57	-1.05
PAX8	paired box	207921_x_	1.59E-15	1.60E-16	-9.59	26.73	-0.97
PAX8	paired box	213917_at	5.77E-15	6.12E-16	-9.34	25.39	-1.09
PAX8	paired box	214528_s_	1.32E-14	1.44E-15	-9.19	24.54	-0.99

Disease Models & Mechanisms • Supplementary information
